# Single‐cell profiling‐guided combination therapy of c‐Fos and histone deacetylase inhibitors in diffuse large B‐cell lymphoma

**DOI:** 10.1002/ctm2.798

**Published:** 2022-05-06

**Authors:** Luqiao Wang, Zijuan Wu, Yi Xia, Xueying Lu, Ji Li, Lei Fan, Chun Qiao, Hairong Qiu, Danling Gu, Wei Xu, Jianyong Li, Hui Jin

**Affiliations:** ^1^ Department of Hematology, Pukou CLL Center, the First Affiliated Hospital of Nanjing Medical University Jiangsu Province Hospital Nanjing China; ^2^ Key Laboratory of Hematology of Nanjing Medical University Nanjing Medical University Nanjing China; ^3^ Jiangsu Key Lab of Cancer Biomarkers, Prevention and Treatment, Collaborative Innovation Center for Personalized Cancer Medicine Nanjing Medical University Nanjing China; ^4^ Singleron Biotechnologies Nanjing China; ^5^ National Clinical Research Center for Hematologic Diseases the First Affiliated Hospital of Soochow University Suzhou China

**Keywords:** combination treatment, diffuse large B‐cell lymphoma, histone deacetylase inhibitor, single‐cell RNA sequencing

## Abstract

**Background:**

Diffuse large B‐cell lymphoma (DLBCL) is the most common subtype of non‐Hodgkin lymphoma. Histone deacetylase inhibitors (HDACis) have been widely applied in multiple tumours, but the expected efficacy was not observed in DLBCL. Therefore, this study is aimed to explore superior HDACis and optimise a relative combinational therapeutic strategy.

**Methods:**

The antitumour effects of the drug were evaluated by Cell Counting Kit‐8 (CCK‐8) assay and apoptosis analysis. Single‐cell RNA sequencing (scRNA‐Seq) was used to analyse the intratumoural heterogeneity of DLBCL cells. Whole‐exome sequencing and RNA sequencing were performed to analyse the genetic and transcriptional features. Western blotting, qRT–PCR, protein array, immunohistochemistry, and chromatin immunoprecipitation assays were applied to explore the involved pathways. The antitumour effects of the compounds were assessed using subcutaneous xenograft tumour models.

**Results:**

LAQ824 was screened and confirmed to kill DLBCL cells effectively. Using scRNA‐Seq, we characterised the heterogeneity of DLBCL cells under different drug pressures, and c‐Fos was identified as a critical factor in the survival of residual tumour cells. Moreover, we demonstrated that combinatorial treatment with LAQ824 and a c‐Fos inhibitor more potently inhibited tumour cells both in vitro and in vivo.

**Conclusion:**

Altogether, we found an HDACi, LAQ824, with high efficacy in DLBCL and provided a promising HDACi‐based combination therapy strategy.

## Graphical abstract

1. LAQ824, a superior histone deacetylase inhibitor (HDACi), effectively kills diffuse large B‐cell lymphoma (DLBCL) cells, especially when combined with a c‐Fos inhibitor.
2. Single‐cell analysis in DLBCL cells treated with a high dose of LAQ824 identified c‐Fos as a critical factor in the survival of residual tumour cells.
3. The acetylation of H3K9 and phosphorylation of H3S10 contribute to increasing c‐Fos under the pressure of LAQ824.

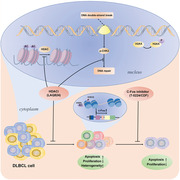

## 1 INTRODUCTION

Diffuse large B‐cell lymphoma (DLBCL) is a highly aggressive lymphoma with considerable heterogeneity.^1^ Although R‐CHOP chemotherapy (rituximab, cyclophosphamide, doxorubicin, vincristine, and prednisone) significantly improves the survival of patients, approximately 30%–40% of patients do not respond or eventually develop refractory disease, which is a dilemma in current clinical practice.^2^, ^3^ Histone deacetylase inhibitors (HDACis) are promising drugs with various biological roles in cell cycle arrest, cell apoptosis, DNA damage, and repair.^4^, ^5^, ^6^ Until recently, several HDACi such as romidepsin, belinostat, and panobinostat, have been applied in clinical trials, showing encouraging performance in peripheral T‐cell lymphoma, cutaneous T‐cell lymphoma, and multiple myeloma.^7^, ^8^, ^9^, ^10^, ^11^, ^12^ However, the limited efficacy of these HDACi against DLBCL has been observed as a single agent.^13^, ^14^ Therefore, there is an urgent need to find a superior HDACi and explore combinational therapeutic strategies to improve the clinical outcomes of patients with DLBCL. Dacinostat (LAQ824) is a hydroxamic acid‐based HDACi that shows extensive enzyme inhibitory activity in the nanomolar range.^15^, ^16^ Recent evidence suggests that LAQ824 may enhance the chemosensitivity of tumour cells by interfering with cell survival and proliferation^17^ and inducing cell apoptosis.^18^ In this study, we found a profound effect of LAQ824 on DLBCL cells. Single‐cell RNA sequencing (scRNA‐Seq) was performed to explore the clonal evolution characteristics of DLBCL cells under treatment pressure with different doses of LAQ824.

The proto‐oncogene c‐Fos forms a heterodimer with the c‐Jun protein, which leads to the formation of the activator protein‐1 (AP‐1) complex, participating in cell proliferation, apoptosis, and DNA damage repair.^19^, ^20^, ^21^ For example, previous studies have revealed the oncogenic role of c‐Fos in the occurrence and development of various malignant tumours, such as cartilage tumours and human oesophageal cancer.^22^, ^23^, ^24^, ^25^ Researchers have also found that inhibition of c‐Fos may eradicate resistance to tyrosine kinase inhibitors in BCR‐ABL‐induced leukaemia.^26^ In our study, c‐Fos was upregulated following treatment with LAQ824 according to scRNA‐seq data. We have also discovered, for the first time, that c‐Fos can be used as an indicator of the sensitivity of HDACi, and the combination of LAQ824 and c‐Fos inhibitors synergistically killed DLBCL cells in vitro and in vivo. Altogether, our findings provide a new perspective for applying HDACi in DLBCL and present potential combinational treatment strategies.

## 2 METHODS

### 2.1 Cell lines

The human DLBCL cell lines OCI‐LY3, OCI‐LY7, U2940, and WILL1 were purchased from Nanjing Kaiji Biotech (Nanjing, China). The human DLBCL cell lines SUDHL‐4 and SUDHL‐10 were purchased from FuHeng Cell Center (Shanghai, China). The human DLBCL cell lines SUDHL‐6, SUDHL‐8, and DB were purchased from Shanghai Zhong Qiao Xin Zhou Biotechnology Co., Ltd. Human DLBCL cell lines CTB1, KIS1, HBL‐1, MEDB1, BJAB, RI‐1, U2932, and FARAGE were purchased from Nanjing Daona Biological Technology Co., Ltd. (Nanjing, China). U2932, HBL‐1, FARAGE, CTB‐1, KIS1, BJAB, RI‐1, SU‐DHL‐4, SU‐DHL‐10, U2940, SU‐DHL‐6, and DB cells were grown in RPMI‐1640 medium (Gibco) supplemented with 10% fetal bovine serum (FBS), L‐glutamine, and penicillin/streptomycin. The WILL1 and SU‐DHL‐8 cell lines were grown in RPMI‐1640 medium supplemented with 20% FBS, L‐glutamine, and penicillin/streptomycin, and the OCI‐LY3, OCI‐LY7, and MEDB1 cell lines were maintained in IMDM (Gibco) complete medium.

### 2.2 Chemical reagents and antibodies

An inhibitor of c‐Fos, difluorobenzocurcumin (CDF), was purchased from BOC Science (NY, USA). T‐5224 (c‐Fos/AP‐1 inhibitor) was purchased from MedChemExpress. The HDACis dacinostat (LAQ824), vorinostat (SAHA), entinostat (MS‐275), belinostat (PXD101), tucidinostat (chidamide), and abexinostat (PCI‐24781) were purchased from Selleck. Antibodies against phospho‐Chk2 (Thr68), phospho‐ATM (Ser1981), c‐Fos, phospho‐histone H2AX (Ser139), cleaved PARP, cleaved caspase‐3, and cleaved caspase‐9 were purchased from Cell Signaling Technology (Danvers, MA, USA). Antibodies against PARP, Caspase‐3, Caspase‐9, BCL‐2, CHK2, and ATM were purchased from Proteintech. Acetyl‐histone H3 antibodies were purchased from Millipore. Anti‐H3K9ac antibody was obtained from ABclonal. Anti‐pH 3Ser10 was obtained from Thermo Fisher Scientific.

### 2.3 Xenograft study

Male nonobese diabetic, severe combined immunodeficiency (NOD SCID) mice (8–12 weeks of age) were selected to inoculate U2932 cells (1*107 cells per animal) subcutaneously through the armpits of the forelimbs. U2932 cells were resuspended in phosphate‐buffered saline (PBS) and then mixed with Matrigel in a volume ratio of 1:1. When the tumour reached a size of 100∼300 mm^3^, the mice were randomly divided into three groups and treated with vehicle, LAQ824 (75 mg/kg), or a combination of LAQ824 and CDF (10 mg/kg). LAQ824 was injected intravenously, once every 5 days, for four consecutive treatments. CDF was injected intraperitoneally daily for 24 days. Tumours and body weights were measured twice a week. Tumour volumes were monitored with callipers and calculated as follows: volume (mm^3^) = (length × width^2^)/2. At the moment of death, tumour tissues were collected for further analyses.

### 2.4 ^18^F‐FLT PET tracer technology for antitumour efficacy evaluation

^18^F‐FLT PET is a sensitive imaging method for displaying high‐grade lymphoma. It has high sensitivity for detecting lymphoma and can effectively detect the proliferation of lymphoma in vivo. As a positron tracer reflecting cell proliferation, ^18^F‐FLT can be applied to positron emission tomography (PET) imaging to observe the proliferation of tumours in the body at the molecular level noninvasively and quantitatively, which improves the specificity of tumour diagnosis and provides a method to detect tumour response to treatment. In this study, we treated the constructed DLBCL model mice under different conditions, injected ^18^F‐FLT intravenously before and on the third day after administration, and performed a static PET scan of the whole body 1 hour later. The radioactive uptake value of each tissue was obtained in partment model (PMOD).

### 2.5 Drug preparation

All drugs were dissolved in dimethyl sulfoxide (DMSO) and stored at −20°C before application. For in vivo injection, LAQ824 was diluted in saline solution with 4% DMSO and 40% PEG, whereas CDF was diluted in RPMI‐1640 medium with 10% FBS.

### 2.6 Immunohistochemistry

Histologic sections from relapsed, refractory, and initially diagnosed DLBCL samples were collected at the Institute of Hematology and Blood Diseases Hospital, Chinese Academy of Medical Science, and stained with c‐Fos antibody. Immunohistochemical (IHC) staining of histological sections from mouse formalin fixed paraffin‐embedded (FFPE) tissues was performed for c‐Fos, CHK2, Ki67, and p‐H3S10. Antibodies used in immunohistochemistry were purchased from Abcam (Abcam, Cambridge, UK).

### 2.7 Statistical analysis

All of the experiments were conducted in triplicate. GraphPad Prism software version 8 was used to statistically analyse the data. *P* values were calculated by Student's *t* test and were considered statistically significant when *P* values were < .05. Spearman's rank correlation coefficient was applied to analyse correlative studies. Predicted IC50 data for LAQ824 in different DLBCL cell lines were accessed through the Genomics of Drug Sensitivity in Cancer (GDSC) database (https://www.cancerrxgene.org/). The IC50 values (Sanger GDSC1) data and c‐Fos expression (Expression Public 21Q1) data in different cell lines were acquired via the DepMap portal (https://depmap.org/portal/). Combination indexes (CIs) were analysed by the Chou Talalay algorithm method.^27^ The CIs for all assessed combinations are shown. Synergism, additive effects, and antagonism were defined as CI<1, CI = 1 and CI>1, respectively.

## 3 RESULTS

### 3.1 HDACi LAQ824 can inhibit growth and promote apoptosis of DLBCL cells

**FIGURE 1:**
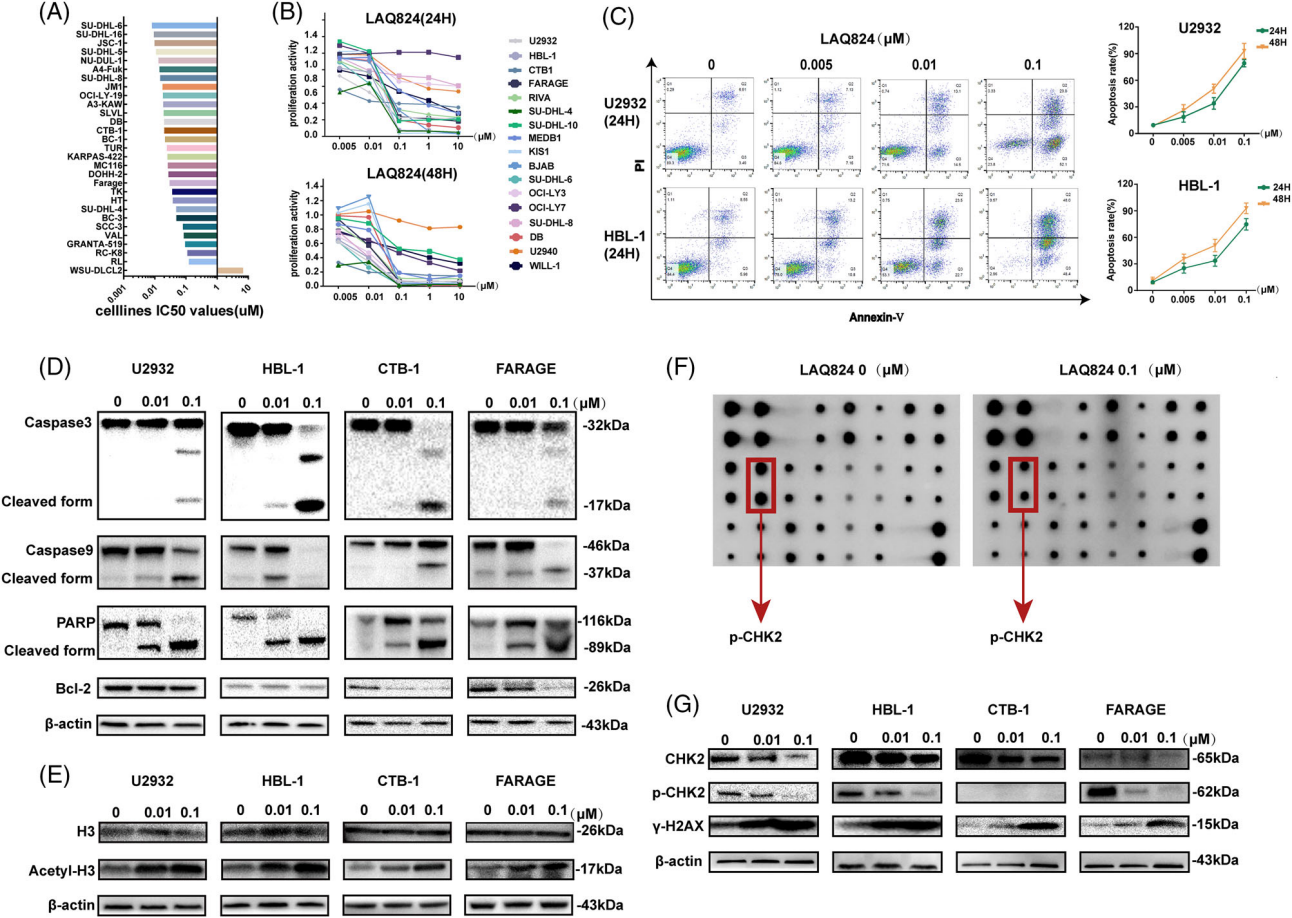
LAQ824 inhibited proliferation and induced apoptosis in diffuse large B‐cell lymphoma (DLBCL) cells. (A) IC50 values of LAQ824 in 29 DLBCL cell lines. We screened out small molecule compounds with low IC50 values in 29 DLBCL cell lines from the genomics of drug sensitivity in the cancer database and found that the histone deacetylase inhibitor dacinostat (LAQ824) inhibited the viability of most DLBCL cell lines. (B) Cell Counting Kit 8 detects the proliferation ability of 17 DLBCL cell lines after 24 and 48 hours of treatments with different concentrations of LAQ824; (C) Apoptosis (annexin V+/DAPI− plus annexin V+/DAPI+) of DLBCL cell lines treated with different concentrations of LAQ824 was detected by flow cytometry. (D) The expression levels of the apoptotic proteins caspase3/9 and PARP, their cleaved form, and the antiapoptotic protein BCL‐2 were detected by western blotting after treatment with different concentrations of LAQ824 for 24 hours. (E) Western blot analysis of the acetylation of H3 in DLBCL cells treated with LAQ824. Total H3 and β‐actin were similarly analysed. (F) The protein array analysed the related protein expression of DLBCL cells after treatment with LAQ824. Representative images of protein array analysis showed that the phosphorylated checkpoint kinase 2 in DLBCL cells treated with LAQ824 was decreased compared to the control group. (G) The array results were further verified by western blotting, and the expression of γH2AX, a marker of DNA double‐strand breaks, was detected in the cells treated with LAQ824. All experiments were performed three times. Data are represented as the mean ± standard deviation. * *P* < .05, ** *P* < .01, *** *P* < .001 versus the control group

First, we screened small molecule compounds that could effectively kill DLBCL cells using the genomics of drug sensitivity in cancer (GDSC; www.cancerRxgene.org) database. Dacinostat (LAQ824), a HDACi with low IC50 values in most DLBCL cell lines, was selected (Figure 1A). To verify the killing effect on DLBCL cells, we detected the proliferation of 17 DLBCL cell lines, including germinal centre B (GCB) and non‐GCB types, under different conditions. Cell Counting Kit 8 (CCK8) assays showed that after LAQ824 treatment, the survival rate of most cell lines was remarkably suppressed (Figure 1B). Chidamide is a selective inhibitor of HDAC1, HDAC2, HDAC3, and HDAC10^28^ and has been approved by the China Food and Drug Administration for clinical application.^29^ We then compared the cytotoxic effects of chidamide and LAQ824 on DLBCL cells. We observed that the killing effect of LAQ824 was dramatically more substantial than that of chidamide at the same concentration (Figure S1A,B). Furthermore, flow cytometric analysis of two DLBCL cell lines (U2932 and HBL‐1) indicated that LAQ824 could induce cell apoptosis (Figure 1C). Concurrently, the expression level of activated apoptotic proteins was increased, while anti‐apoptotic proteins decreased with LAQ824 treatment (Figure 1D). LAQ824 was reported to be an effective HDACi.^18^ We also detected the acetylation level of histone H3 (H3ac) and found that H3ac was upregulated after drug treatment (Figure 1E). In addition, the protein array results demonstrated that the expression of phosphorylated checkpoint kinase 2 (p‐Chk2), which is critical to the process of DNA damage and repair,^30^ was inhibited after LAQ824 treatment, as confirmed by western blotting (Figure 1F,G). The phosphorylation of histone H2AX at Ser 139 (γH2AX) is a marker of DNA double‐strand breaks (DSBs).^31^ LAQ824 can induce upregulation of γH2AX expression (Figure 1G). These results indicated that LAQ824 upregulated histone acetylation and exerted a killing effect on DLBCL cells by inhibiting p‐Chk2 expression and weakening the DNA repair capacity.

### 3.2 Single‐cell profiling of DLBCL cells under the drug pressure of LAQ824

**FIGURE 2:**
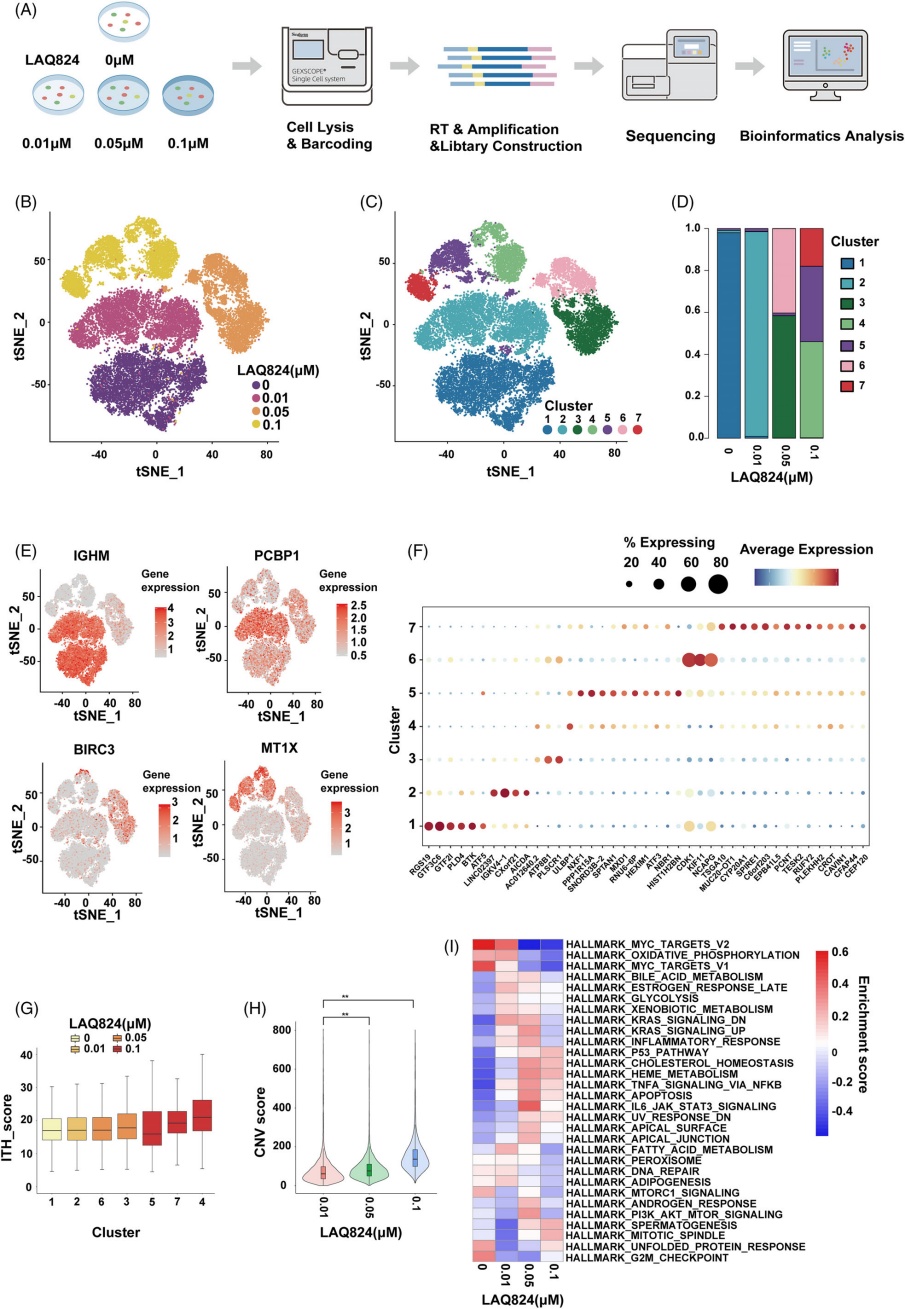
Single‐cell RNA sequencing (scRNA‐Seq) analysis of diffuse large B‐cell lymphoma (DLBCL) cells treated with LAQ824. (A) Schematic of the scRNA‐Seq experiment where the DLBCL cell line U2932 was treated with multiple concentrations (0/0.01/0.05/0.1 μM) of LAQ824 in vitro for 24 hours and subjected to the GEXSCOPE^®^ platform (Singleron). (B–D) t‐stochastic neighbor embedding (tSNE) visualisation of 11,420 cells labelled by different concentrations of LAQ824 (n = 4) and by different cell subtypes (n = 7), and the proportion and distribution of each subgroup in different samples are shown. (E) Gene expression levels of marker genes associated with concentrations of LAQ824 that were identified by clustering and visualised with tSNE. (F) Dot plot illustrating the differences in gene expression in different cell subgroups of each group of samples. (G) Intratumoural heterogeneity analysis of different cell subpopulations. (H) The copy number variation score for DLBCL cells treated with different concentrations of LAQ824 was analysed. (I) Gene set variation analysis using hallmark gene sets was performed on scRNA‐Seq data. Gene set variation analysis enrichment scores calculated by each gene set of single cells using log2 (UMI + 1) data are shown

We performed scRNA‐seq on the DLBCL cell line U2932, which was treated with different concentrations of LAQ824 (Figure 2A). After quality control and unsupervised clustering, we obtained the transcriptomes of 11,420 cells consisting of four groups with varying doses of LAQ824 and further differentiated them into seven clusters (Figure 2B,C). Simultaneously, the distribution and proportion of each cluster in the different samples were analysed (Figure 2D), revealing that the heterogeneity of residual tumour cells was gradually enhanced with an increasing dose of LAQ824. Dose‐dependent transcriptional changes in DLBCL cells were observed after treatment with LAQ824 (Figure 2E and Figure S2A,B). Furthermore, we used differentially expressed gene signatures to assign cell identities to these clusters (Figure 2F and Figure S2C,D). In addition, the expression of B‐cell‐related specific markers in DLBCL cells treated with LAQ824 was found to be decreased (Figure S2E). Intratumoural heterogeneity analysis of different cell subpopulations showed relatively enhanced levels of clusters 4, 5, and 7 (Figure 2G). A higher copy number variation (CNV) score and mutation frequency were discovered in DLBCL cells treated with .1 μM LAQ824 (Figure 2H and Figure S2F). Subsequent gene set variation analysis was conducted to better explore the activated pathways in different samples (Figure 2I).

### 3.3 c‐Fos is upregulated in DLBCL cells treated with a high dose of LAQ824 at the single‐cell level

**FIGURE 3:**
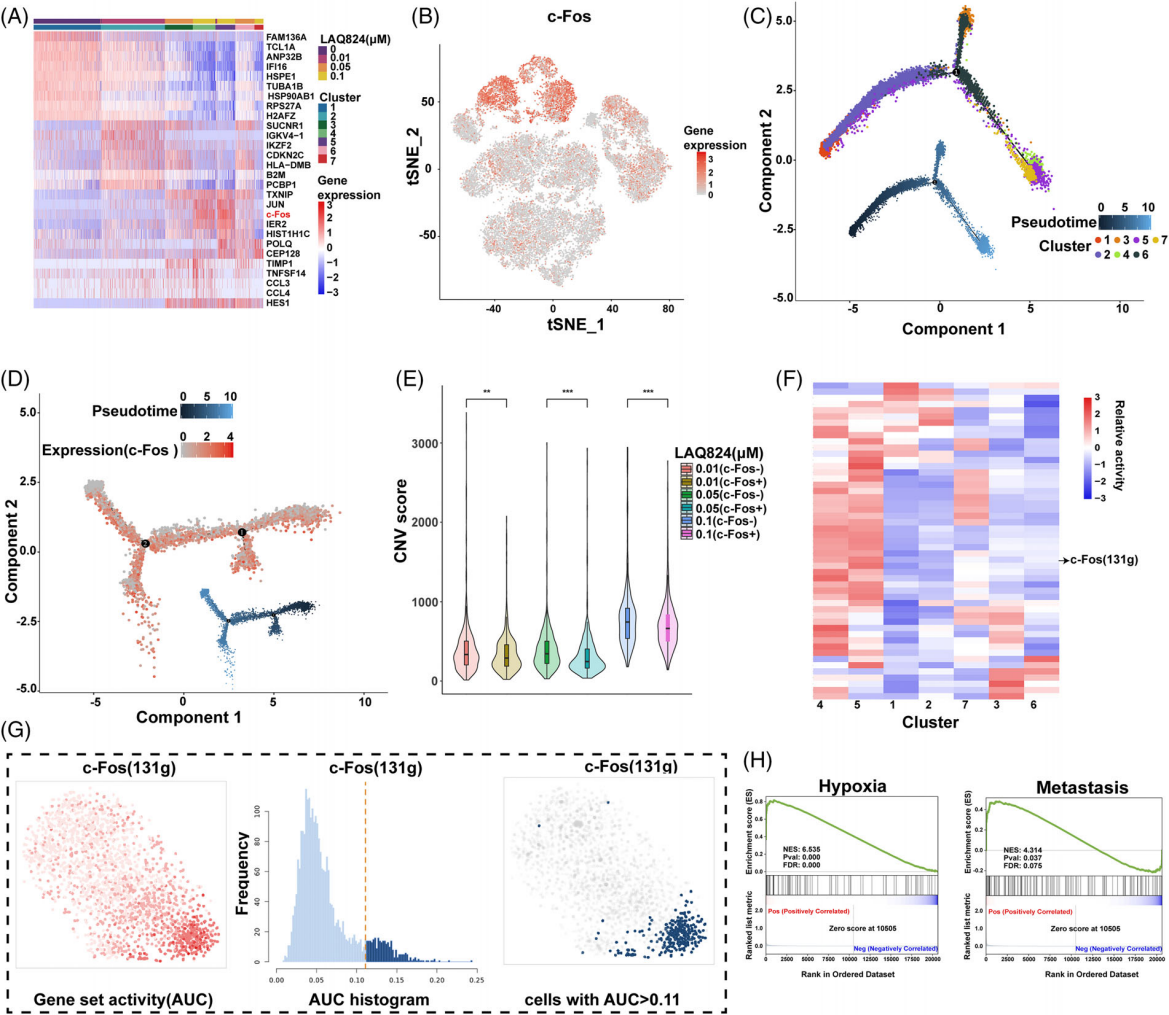
LAQ824 induced upregulation of c‐Fos expression in diffuse large B‐cell lymphoma (DLBCL) cells. (A) Heatmap showing the differentially expressed genes in different samples and each cluster (labelled by different samples and each cluster as in Figure 1B,C), and c‐Fos was significantly upregulated in the .1 μM LAQ824 treatment group. (B) The t‐stochastic neighbor embedding (tSNE) plot shows the expression level of c‐Fos in different samples. (C) Pseudo‐time trajectories developed through Monocle2 analysis for DLBCL cells treated with different concentrations of LAQ824. (D) Monocle‐based pseudo‐time ordering predicts that DLBCL cells exposed to .1 μM LAQ824 move along different trajectories, and the expression pattern of c‐Fos is plotted along the pseudo‐time axis. (E) The copy number variation score for different c‐Fos‐positive and c‐Fos‐negative samples was analysed. (F) Using SCENIC, we identified transcription factors (TFs) unique to each cluster. Heatmap of the *t* values of area under the curve (AUC) scores of expression regulation by transcription factors of each cluster, as estimated using SCENIC. (G) SCENIC analysis predicts TFs such as c‐Fos as central hubs in DLBCL cells treated with LAQ824. TF regulon activities were quantified using AUCell. (H) Gene Set Enrichment Analysis (GSEA) shows the top enriched pathways in a cluster with high expression of c‐Fos. NES denotes the normalised enrichment score. *P < 0.05, **P < 0.01, ***P < 0.001 versus the control group

When the concentration of LAQ824 reached .1 μM, c‐Fos was significantly upregulated (Figure 3A and Figure S3A,B), especially in clusters 4 and 5 (Figure 3B and Figure S3C). Simultaneously, we performed bulk RNA‐seq, and the trend of c‐Fos expression was consistent with the scRNA‐Seq (Figure S3D). These results suggest that c‐Fos may be related to the increased heterogeneity of DLBCL cells and the reaction to LAQ824. To assess the expression characteristics of c‐Fos, cells treated with .1 μM LAQ824 were resorted into 10 clusters (Figure S4A), and the distribution and proportion of each subgroup were analysed (Figure S4B). Specific marker genes for each cluster were visualised through a dot plot and heatmap (Figure S4C–E). As expected, c‐Fos was highly expressed in most clusters (Figure S4F). Pseudotime analysis was performed to determine the evolutionary trajectory under different drug pressures (Figure 3C and Figure S4G,H). Interestingly, pseudo‐time reconstruction analysis of residual tumour cells revealed the loss of c‐Fos expression at the end of this trajectory. The hierarchy showed that c‐Fos‐expressing cell subgroups eventually evolved into c‐Fos‐negative cells under the action of drugs (Figure 3D), and the CNV score and mutation frequency of c‐Fos‐negative clusters were higher than those of c‐Fos‐positive clusters (Figure 3E). To further explore the role of c‐Fos, we used SCENIC to predict the complex regulatory network in cells treated with LAQ824, and genes regulated by c‐Fos were highly upregulated in clusters 4 and 5 (Figure 3F,G). Gene Set Enrichment Analysis (GSEA) was also performed, and we discovered enriched hypoxia and metastasis pathways in groups with higher c‐Fos expression (Figure 3H).

### 3.4 Upregulated c‐Fos promotes the survival of residual tumour cells

**FIGURE 4:**
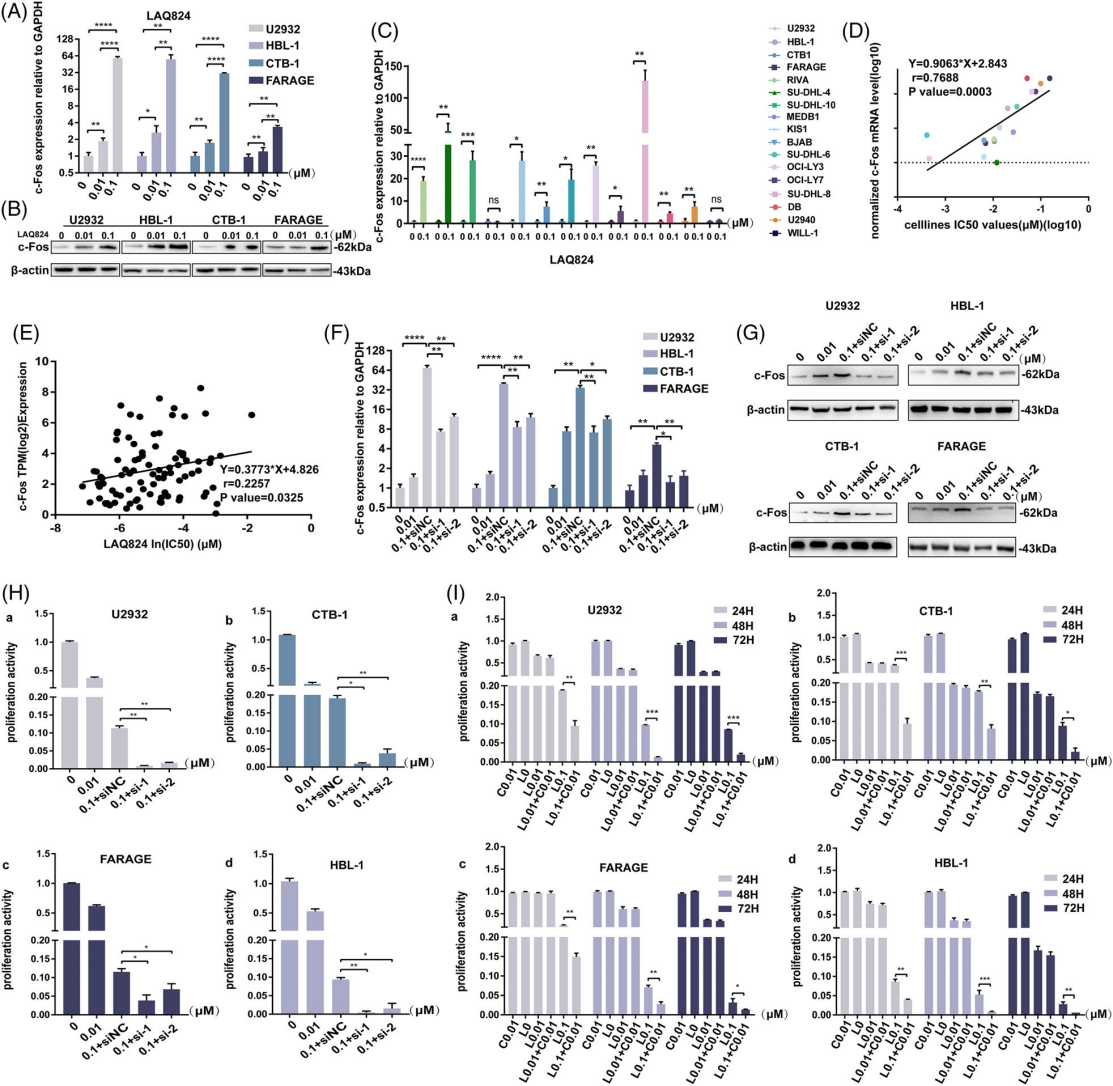
Overexpression of c‐Fos helps diffuse large B‐cell lymphoma (DLBCL) cells resist the pressure of LAQ824. (A and B) The expression levels of c‐Fos mRNA and protein in DLBCL cell lines treated with different concentrations of LAQ824. (C, D) qRT–PCR results showed that LAQ824 induced upregulation of c‐Fos mRNA expression in other DLBCL cell lines. Correlation between c‐Fos mRNA expression level and IC50 of LAQ824 in different DLBCL cell lines. (E) Correlation analysis of IC50 values of LAQ824 (Sanger GDSC1) and c‐Fos expression levels (Expression Public 21Q1) in multiple myeloma (MM), acute myeloid leukaemia (AML), and lymphoma cell lines. (F, G) qRT–PCR and immunoblot analysis of c‐Fos in U2932, HBL1, CTB, and FARAGE cell lines at 48 hours after transfection with a siRNA targeting c‐Fos or a control siRNA and treated with LAQ824 (0.1 μM) simultaneously. β‐actin served as controls. (H) Cell Counting Kit 8 analysis of proliferation activity in U2932, HBL1, CTB, and FARAGE cell lines transfected as in (D) and treated with LAQ824 (0.1 μM). (I) LAQ824 (0/0.01/0.1 μM) combined with a c‐Fos inhibitor (0.01 μM CDF) synergistically inhibited proliferation in different DLBCL cell lines. C: CDF (0.01 μM). All experiments were performed three times. Data are represented as the mean ± SD. * *P* < 0.05, ** *P* < 0.01, *** *P* <0.001 versus the control group. NS, not statistically significant

qRT–PCR and western blotting detected the upregulation of c‐Fos in four DLBCL cell lines under increased concentrations of LAQ824 (Figure 4A,B), and we verified these results in other DLBCL cell lines (Figure 4C). In addition, we analysed the IC50 values of LAQ824 and found that it was highly positively correlated with c‐Fos expression (Figure 4D). Further analysis revealed no significant differences between the ABC and GCB DLBCL cell lines (Figure S5A). We also calculated the correlation between the IC50 and c‐Fos levels in haematological diseases, including multiple myeloma, acute myeloid leukaemia, and lymphoma, using an online database, and the results showed a positive correlation (Figure 4E). Subsequent results showed that synchronous c‐Fos‐siRNA and LAQ824 treatments significantly inhibited the expression of c‐Fos at the mRNA and protein levels (Figure 4F,G). The suppression of cell viability was enhanced by this combination treatment (Figure 4H). We combined the c‐Fos inhibitors T‐5224 and CDF with LAQ824, and CCK8 assays showed a more substantial killing effect on different DLBCL cell lines than LAQ824 monotherapy (CI<1) (Figure 4I, Figure S5B, and Table S1). The synergistic effect stronger at doses .1 μM LAQ824 than .01 μM may be due to the low expression level of c‐Fos at LAQ824 .01 μM.

### 3.5 Characteristics of gene mutations in DLBCL related to LAQ824 IC50 and c‐Fos expression

**FIGURE 5:**
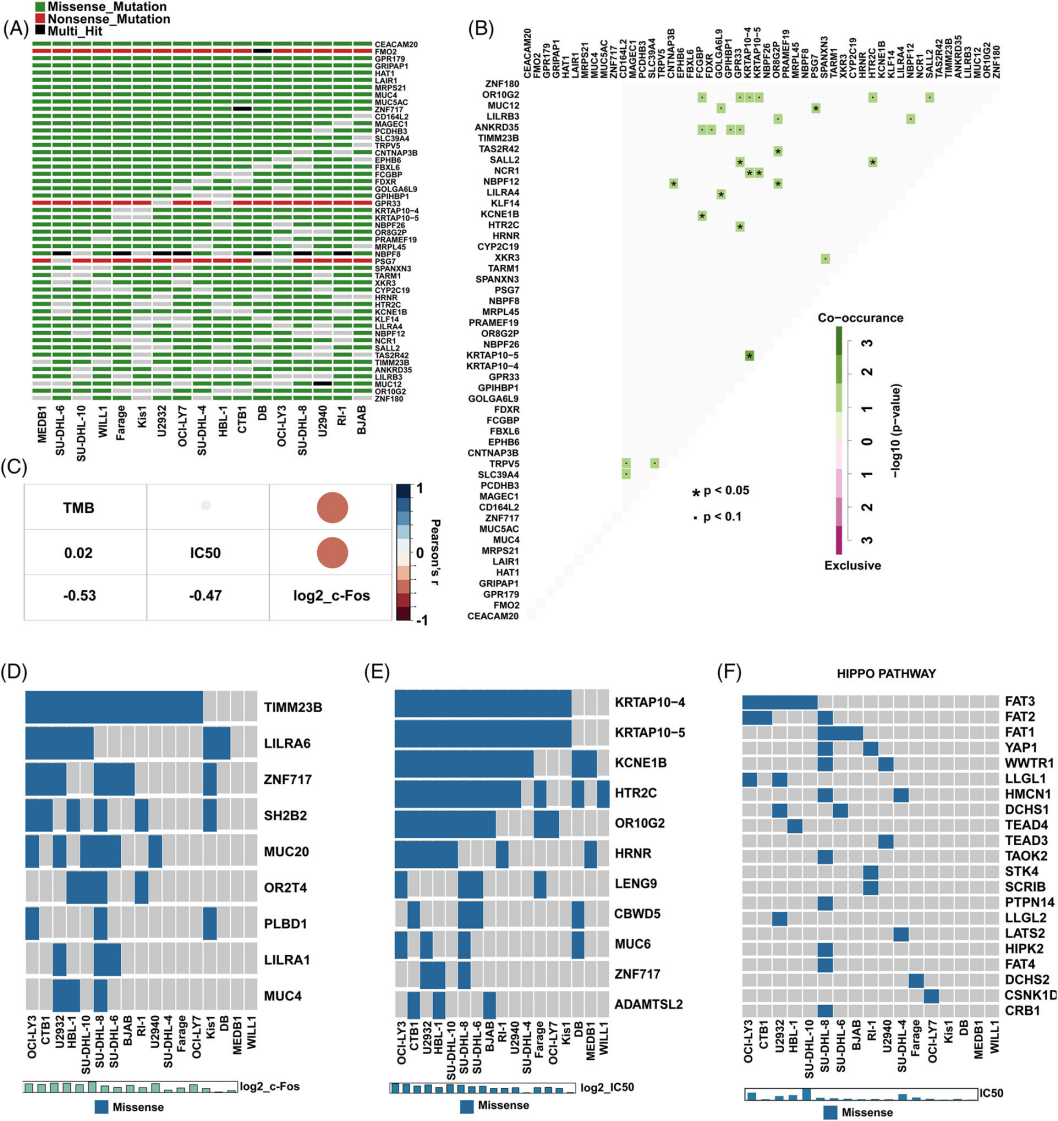
Whole‐exome sequencing analysis of the association between genetics and drug response. (A) Oncoplot for the top 50 mutated genes in the 17 diffuse large B‐cell lymphoma (DLBCL) cell lines. Each row represents a gene, and each column represents the samples. (B) Mutually exclusive and co‐occurring gene pairs in DLBCL cell lines are displayed as a triangular matrix. Green indicates a tendency towards co‐occurrence, whereas pink indicates a tendency towards exclusiveness. (C) The IC50 values of LAQ824/fold change of c‐Fos mRNA in DLBCL cell lines treated with LAQ824 and tumour mutation burden (TMB) in DLBCL cell lines were correlated using Pearson's *r* correlation coefficient, indicated by the numbers in the heatmap. (D) Oncoplot plot showing the relationship between the fold change of c‐Fos mRNA in DLBCL cell lines treated with LAQ824 and mutated genes. (E) Oncoplot plot showing the relationship between the IC50 values of LAQ824 and mutated genes. (F) Mutated pathways involving genes associated with Hippo signalling in DLBCL cell lines with relatively high IC50 values of LAQ824

Furthermore, we performed whole‐exome sequencing on 17 DLBCL cell lines, and *CEACAM20*, *FMO2*, *GPR179*, *GRIPAP1*, and *HAT1* were identified as the most frequent mutations (Figure 5A). The combination patterns of multiple somatic mutations may reveal the functional relationships between genes in tumorigenesis and screening therapeutic targets.^32^ Thus, we analysed the gene sets mutated in either a mutually exclusive or co‐occurring manner, such as GPR33/SALL2 and PSG7/MUC12 (co‐occurring, *P* value < 0.05) (Figure 5B). As an emerging biomarker, tumour mutational burden (TMB) plays a key role in predicting the efficacy of tumour immunotherapy.^33^ Therefore, we analysed the correlations between TMB and IC50 or c‐Fos expression in DLBCL cells treated with LAQ824, and TMB was negatively associated with c‐Fos, while it had no significant association with IC50 (Figure 5C). To further explore the correlation of mutated genes, pathways, IC50, and c‐Fos expression, we divided the samples into two groups according to the presence or absence of mutations. We observed the upregulation of c‐Fos expression in cells with mutations such as *TIMM23B* and *LILRA6* (Figure 5D). Meanwhile, cells with mutations such as *KCNE1B*, *HTR2C*, and *OR10G2* exhibited higher IC50 values (Figure 5E). We also found that cells with higher IC50 values carried more frequent mutations in genes related to the Hippo pathway (Figure 5F).

### 3.6 The acetylation of H3K9 and phosphorylation of H3S10 contribute to increasing c‐Fos under LAQ824 pressure

**FIGURE 6:**
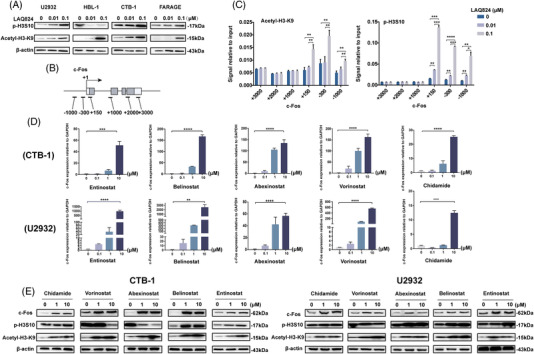
The mechanism by which LAQ824 induces c‐Fos expression and the expression of c‐Fos in other histone deacetylase inhibitors (HDACis). (A) Western blot analysis of the protein expression levels of p‐H3S10 and acetyl‐H3K9 in diffuse large B‐cell lymphoma (DLBCL) cell lines treated with different concentrations of LAQ824. (B) Schematic diagram of different positions of the c‐Fos gene detected in chromatin immunoprecipitation (ChIP) analysis. (C) The CUT&RUN Assay Kit was used to detect the phosphorylation of H3S10 and the acetylation of H3K9 at different positions of the c‐Fos gene in DLBCL cells (U2932) treated with different concentrations of LAQ824. (D) qRT–PCR detected the mRNA expression level of c‐Fos in DLBCL cell lines of ABC type (U2932) and GCB type (CTB1) treated with different HDACis. (E) Western blot analysis of the protein expression levels of ABC‐type (U2932) and GCB‐type (CTB1) DLBCL cell lines treated with different HDAC inhibitors. All experiments were performed three times. Data are represented as the mean ± standard deviation. * *P* < 0.05, ** *P* < 0.01, *** *P* < 0.001 versus the control group

Previous studies have found that the expression of c‐Fos is closely related to lysine 9 acetylation (H3K9ac) and serine 10 phosphorylation of histone H3 (p‐H3S10).^34^, ^35^, ^36^ H3K9ac increased in a dose‐dependent manner after LAQ824 treatment, and p‐H3S10 showed the same trend in most DLBCL cell lines except for HBL‐1, which may be influenced by other pathways (Figure 6A). The chromatin immunoprecipitation (ChIP) assay revealed that H3S10 was remarkably phosphorylated at +150, while H3K9 was acetylated at promoter −300 (Figure 6B,C). These results suggest that the acetylation of H3K9 and phosphorylation of H3S10 induced by LAQ824 may be related to the upregulation of c‐Fos.

### 3.7 The expression of c‐Fos could also be upregulated by other HDACi

To explore whether other HDACis can induce the upregulation of c‐Fos, five HDACi (entinostat, belinostat, abexinostat, vorinostat, and chidamide) that have been applied clinically or undergoing clinical trials were selected. The expression of c‐Fos was significantly increased by treatment with all five HDACis (Figure 6D). In addition, increased expression of p‐H3S10 and H3K9ac was observed, although the expression of p‐H3S10 in the CTB1 cell line showed the opposite trend after abexinostat and vorinostat treatment (Figure 6E). However, treatment with other drugs, such as BCL2i, did not result in a consistent change (Figure S5C). Taken together, these results indicate that the upregulation of c‐Fos may be specific to HDACi treatment in DLBCL.

### 3.8 LAQ824 and c‐Fos inhibitor synergistically suppress DLBCL cell growth in vivo

**FIGURE 7:**
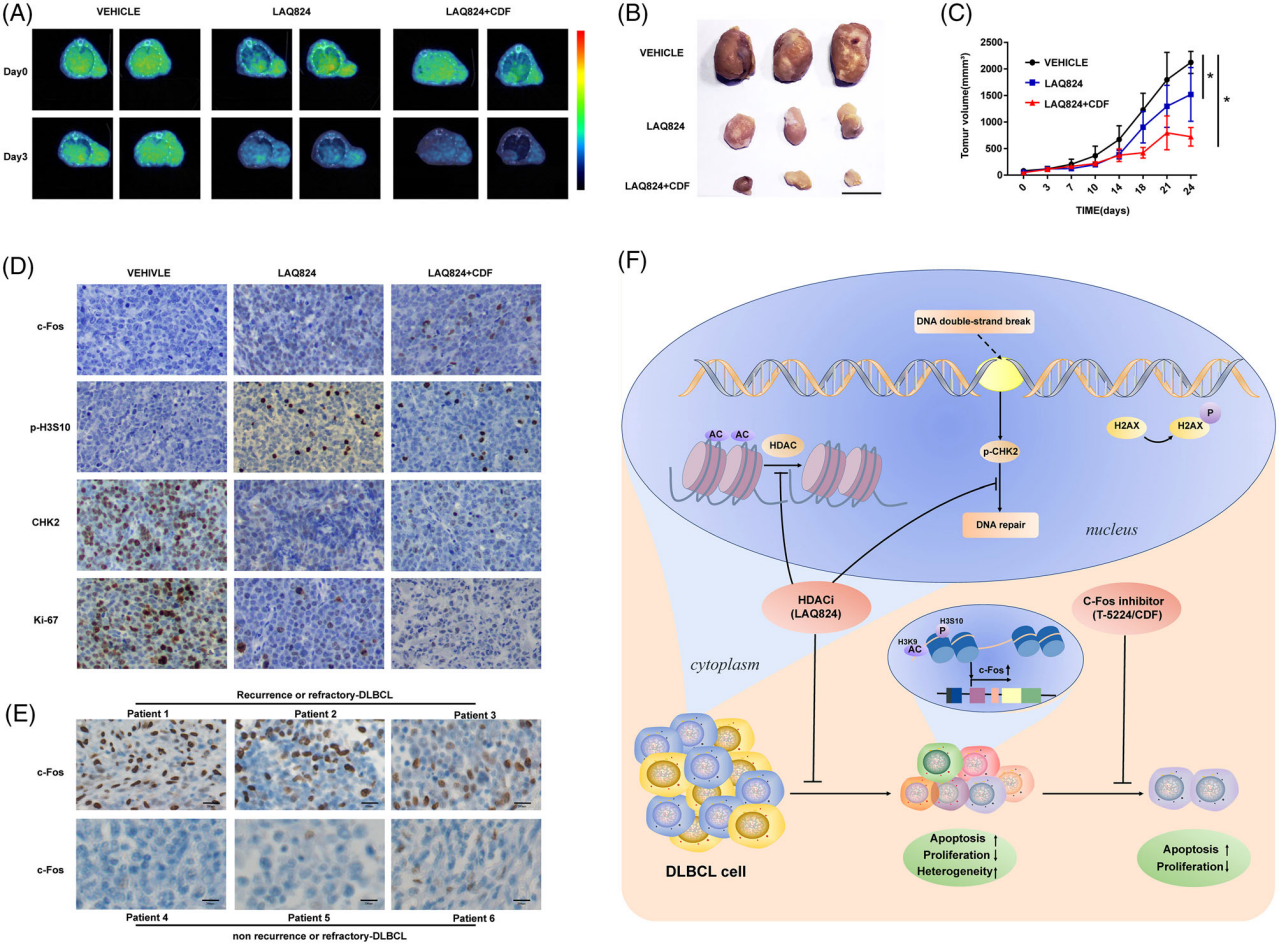
LAQ824 and c‐Fos inhibitor synergistically inhibit diffuse large B‐cell lymphoma (DLBCL) growth in vivo. (A) ^18^F‐FLT PET images showed that nonobese diabetic, severe combined immune deficiency (NOD SCID) mice transplanted with the DLBCL cell line U2932 were treated with LAQ824 (75 mg/kg, once every 5 days), LAQ824 and CDF (10 mg/kg, once a day) combined with vehicle. (B) Illustration of the tumours in each group described above that were removed on day 24. Magnification bar, 10 mm. (C) Tumour volume in mice treated with vehicle only (black), LAQ824 (blue), or LAQ824 in combination with difluorobenzocurcumin (CDF) (red). Data are represented as mean ± SD (n = 6 mice per group, significant vs. vehicle group; * *P* < .05, ** *P* < .01, *** *P* < .001). (D) Tumour tissue sections were subjected to immunochemistry for c‐Fos, checkpoint kinase 2, p‐H3S10, and Ki67 expression (original magnification 200×, scale bar = 100 μm). (E) Representative immunohistochemical (IHC) staining for c‐Fos in initially diagnosed and relapsed or refractory DLBCL patients (original magnification 400×, scale bar = 200 μm). (F) Graph picturing the potential mechanism of combinatorial treatment with LAQ824 and a c‐Fos inhibitor to synergistically inhibit DLBCL cell growth

We further assessed the activity of LAQ824 and the c‐Fos inhibitor CDF in a mouse xenograft model using 18F‐FLT PET and tumour volume analysis. LAQ824 delayed tumour growth, and its effect was dramatically enhanced when combined with CDF (Figure 7A–C). IHC results showed that p‐H3S10 and c‐Fos were upregulated, while CHK2 and Ki67 were reduced in tumours from U2932 xenografts treated with LAQ824. CHK2 and Ki67 were further downregulated by combination treatment with CDF (Figure 7D). Furthermore, we found that c‐Fos levels in R/R DLBCL patients were abnormally elevated compared to those in newly diagnosed patients (Figure 7E). Overall, these results indicate the potential value of LAQ824 and its combined use with a c‐Fos inhibitor in DLBCL.

## 4 DISCUSSION

Considering the beneficial effect of HDACi in DLBCL, we examined the underlying reasons and explored a superior HDACi.^37^, ^38^ Previous studies have reported the activity of LAQ824 against myeloma and acute myeloid leukaemia in vitro and in vivo,^4^, ^39^, ^40^ but its effect on DLBCL is still unknown. In this study, we demonstrated the influential role of LAQ824 in DLBCL cells by affecting DNA damage repair, cell proliferation, and apoptosis. Although our results showed that LAQ824 could inhibit the survival of DLBCL cells, further study of residual tumour cells that were barely killed might help probe the poor efficacy of HDACi monotherapy in clinical application. Thus, we explored the characteristics of tumour cells after treatment with HDACi at the single‐cell level to offer an HDACi‐based combination therapy strategy for DLBCL.

The heterogeneity and clonal evolution of haematological tumours have created obstacles and challenges in clinical diagnosis and treatment. The emergence and maturity of scRNA‐Seq technology provide unprecedented resolution for analysing the pathophysiology of human diseases and offer clues for optimal individualised management of patients. A recent study identified the biological heterogeneity of DLBCL cells at the single‐cell level, which provided opportunities for therapeutic targeting.^41^ Xue et al.^42^ illustrated the cell‐intrinsic events through scRNA‐Seq that are sufficient for a rapid, multifactorial, and nonuniform adaptive process that limits the therapeutic potential of drug inhibition. The clinical efficacy of a single HDACi is limited in DLBCL, and resistance to HDACis may be related to the upregulation of P21 and P27 or the activation of the B‐cell receptor (BCR) pathway.^13^, ^14^ However, the resistance mechanism of HDACis in DLBCL is complicated, and candidate biomarkers that mediate therapeutic responsiveness and drug resistance are urgently needed. In this study, we analysed the single‐cell transcriptome profile of DLBCL cells and verified cellular heterogeneity after LAQ824 treatment. c‐Fos, a key factor, was screened and found to be upregulated under drug pressure and to sustain the survival of residual tumour cells. The contribution of c‐Fos to malignant behaviour indicates its potential significance in disease recurrence and refractory disease. Our data demonstrated that the expression of c‐Fos is increased after HDACi treatment, including LAQ824 and the other five HDACi that are applied clinically or in clinical trials. In addition, c‐Fos was highly correlated with the IC50 of LAQ824 in DLBCL and other haematological diseases. Other inhibitors, such as BCL2i, were also applied in our study and the results indicated the specific resistance role of c‐Fos with HDACi treatment in DLBCL, which needs to be further confirmed. Growing evidence has shown that histone modifications are related to the induction of major histocompatibility complex (MHC) class I and II genes.^43^, ^44^ Consistent with a previous study, we found that treatment of DLBCL cells with LAQ824 also results in an increase in human leukocyte antigen (HLA)‐A expression, which extends the rational basis for using HDACis in combinatorial therapy.

Furthermore, our data elucidated the function of LAQ824 and its enhanced curative effect in combination with c‐Fos inhibitors. These results demonstrate the mechanistic basis for therapeutic targeting of DLBCL and preclinical evidence. In addition, c‐Fos levels in R/R DLBCL patients were higher than those in newly diagnosed patients. Therefore, c‐Fos could be used as a prognostic biomarker. However, this result needs to be verified in a larger cohort of patients.

## 5 CONCLUSIONS

In this project, we screened HDACi, LAQ824, which can effectively kill DLBCL cells and explored its molecular mechanisms. The core regulator c‐Fos was elucidated for the first time to facilitate the survival of residual tumour cells and thus contribute to refractory or recurrent DLBCL. Monitoring the dynamic expression of c‐Fos might provide an early warning for intervention in patients who have received HDACi treatment. Our research offers a new perspective for understanding the unsatisfactory effect of HDACis and represents a promising HDACi‐based combination therapy strategy for DLBCL.

## CONFLICT OF INTEREST

The authors declare that they have no conflict of interest.

## Supporting information

Figure S1

Figure S2

Figure S3

Figure S4

Figure S5

Supporting Information
