# Supplementary figures and images for: Single‐cell profiling‐guided combination therapy of c‐Fos and histone deacetylase inhibitors in diffuse large B‐cell lymphoma
Source: Clin Transl Med. 2022 May 6;12(5):e798. doi: 10.1002/ctm2.798 (PMC9076017; doi:10.1002/ctm2.798)

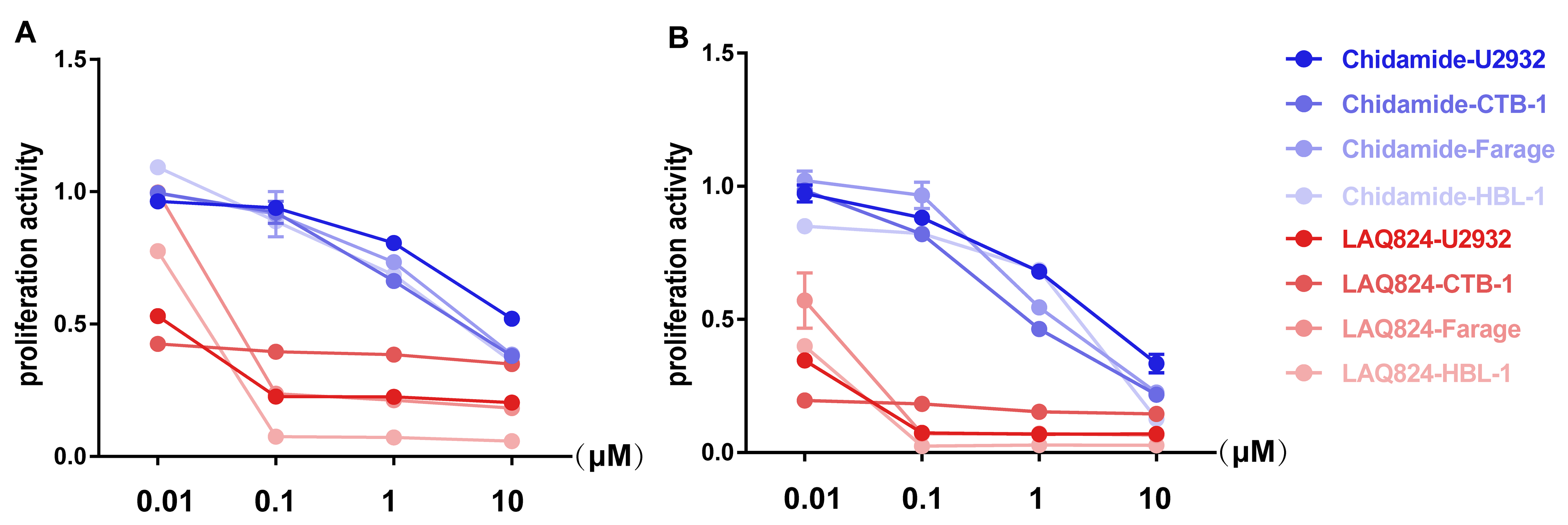

Supplement: Supplementary file 1 — Figure S1 [file CTM2-12-e798-s002.tif]

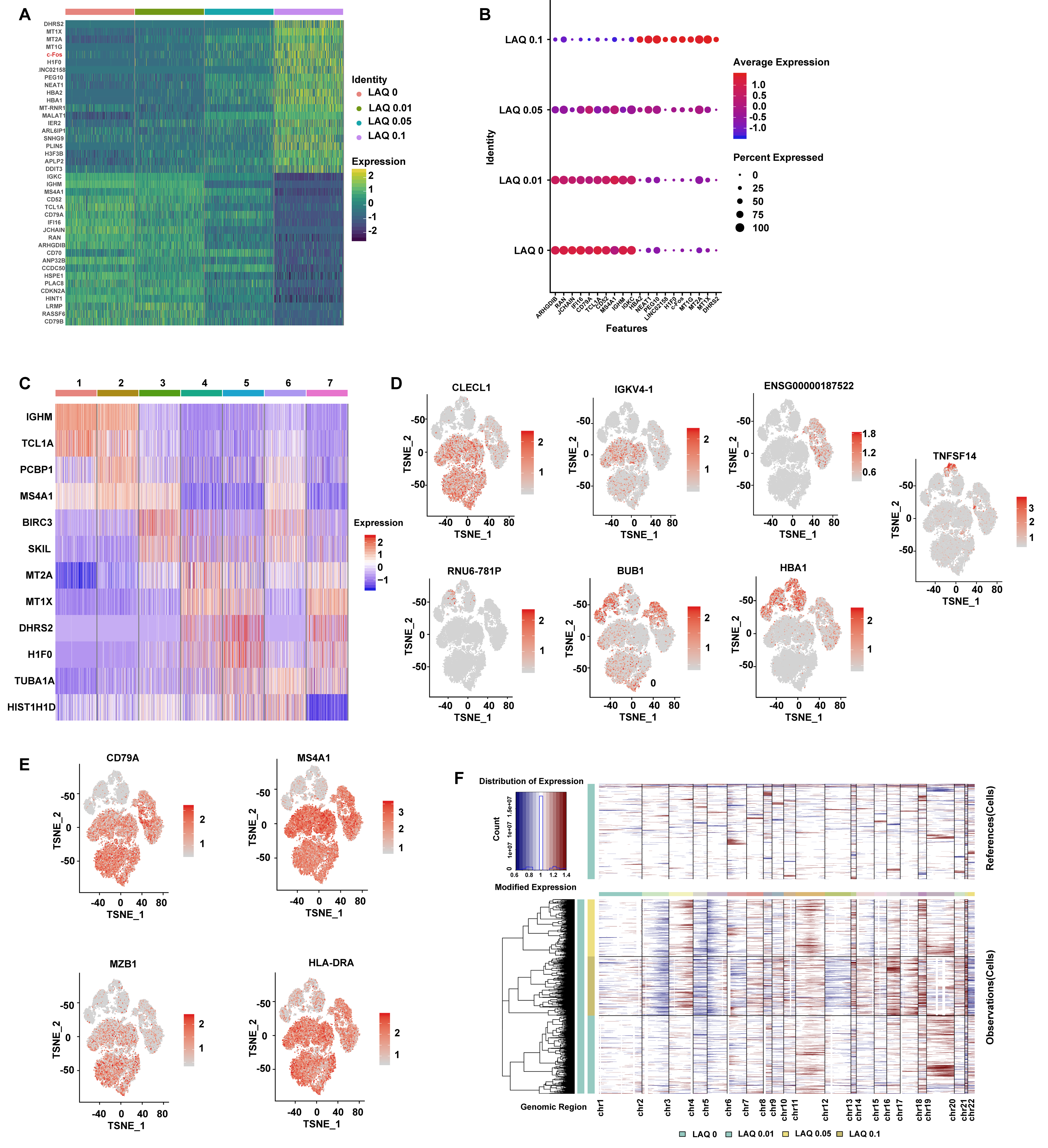

Supplement: Supplementary file 2 — Figure S2 [file CTM2-12-e798-s003.tif]

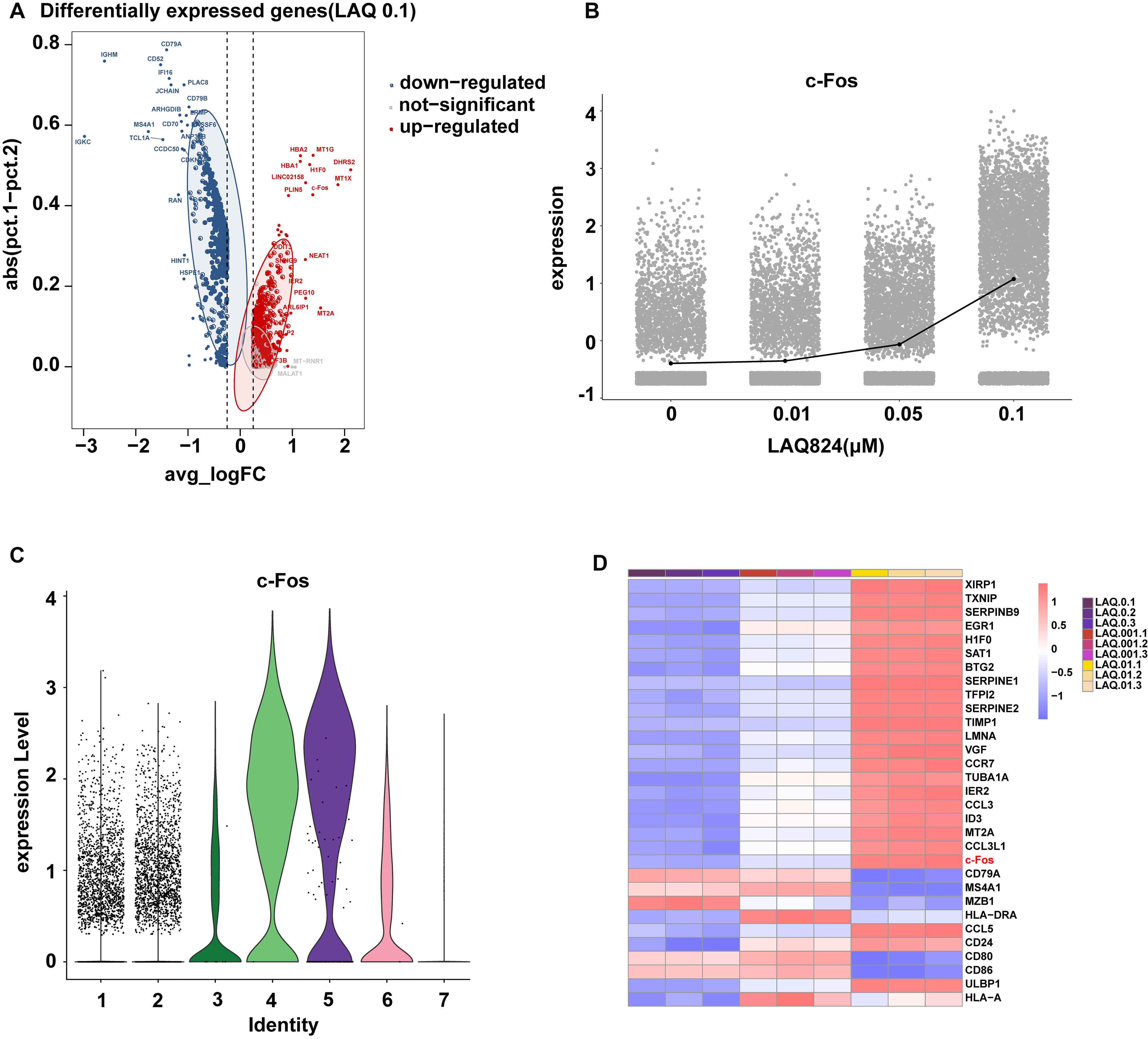

Supplement: Supplementary file 3 — Figure S3 [file CTM2-12-e798-s005.tif]

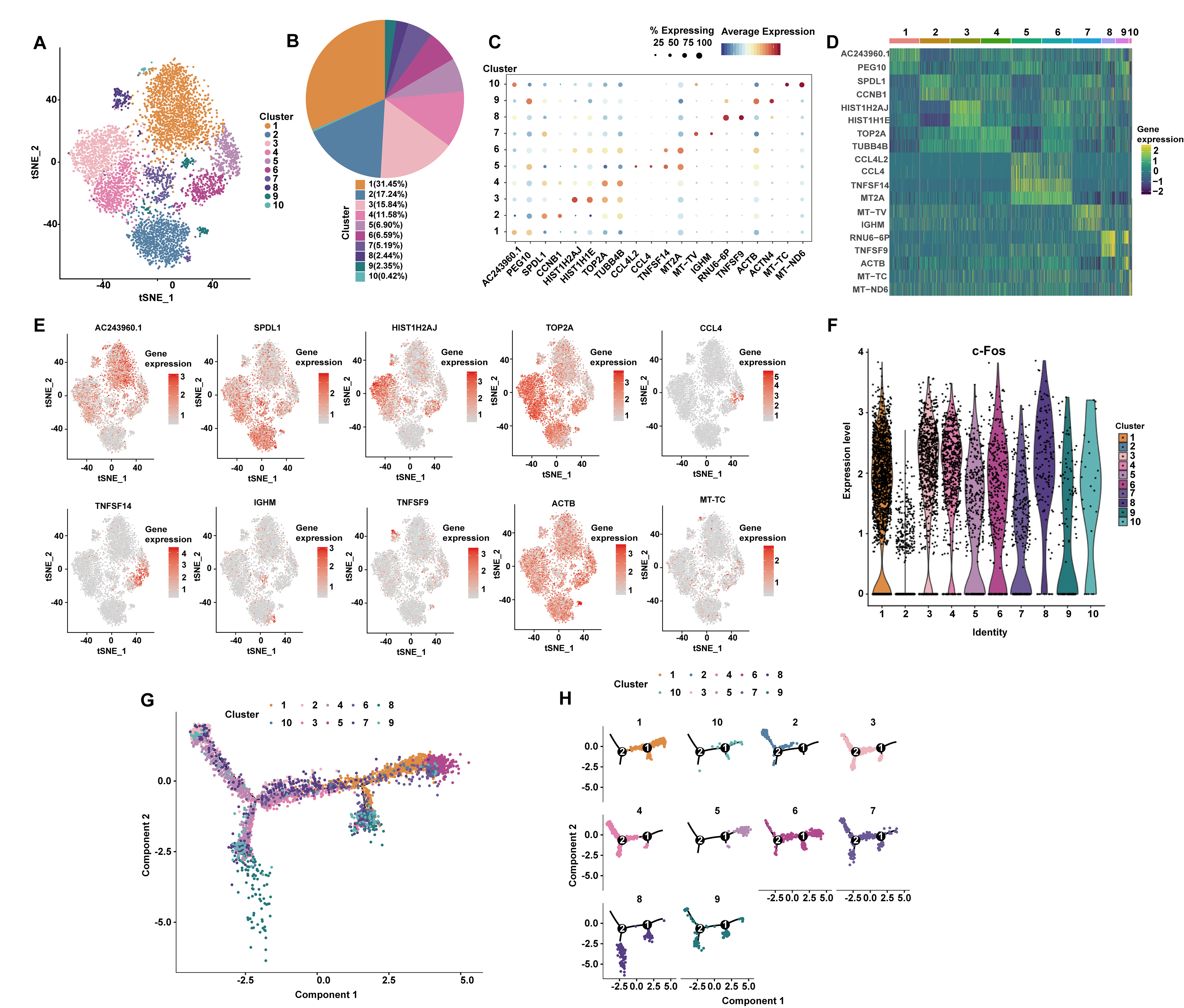

Supplement: Supplementary file 4 — Figure S4 [file CTM2-12-e798-s006.tif]

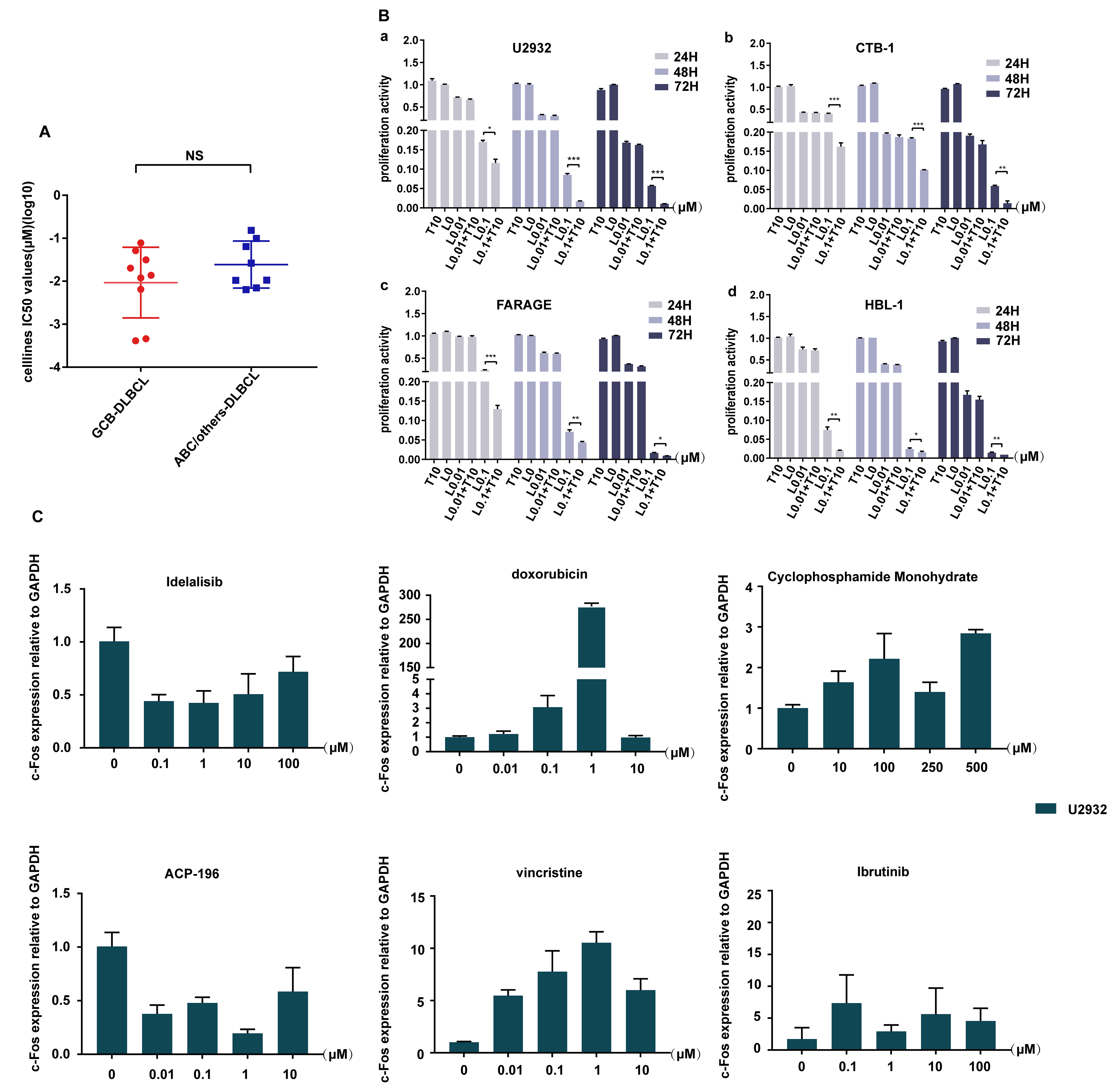

Supplement: Supplementary file 5 — Figure S5 [file CTM2-12-e798-s004.tif]
