# Supplementary material for: Single‐cell profiling‐guided combination therapy of c‐Fos and histone deacetylase inhibitors in diffuse large B‐cell lymphoma
Source: Clin Transl Med. 2022 May 6;12(5):e798. doi: 10.1002/ctm2.798 (PMC9076017; doi:10.1002/ctm2.798)
Supplement: Supplementary file 6 — Supporting Information [file CTM2-12-e798-s001.docx]

**Supplementary Materials**

**Cellular proliferation and apoptosis**

Cell proliferation ability was analyzed by Cell Counting Kit-8 (CCK8). DLBCL cells treated under different conditions were seeded in 96-well plates with 3 replicate wells in each group, and 10ul CCK8 (ApexBio, USA) was added to each well under dark conditions, incubated for 2-4 hours, and the absorbance value was read at 450nm wavelength.

DLBCL cells were treated with different concentrations of LAQ824 or vehicle for 24h and 48h, and apoptosis was analyzed by flow cytometry with FITC-annexin V (early apoptosis) and PI (late apoptosis) using the apoptosis detection kit from FcMACS according to the manufacturer’s instructions. The total apoptosis rate includes early apoptosis (Annexin V+/PI-) and late apoptosis (Annexin V+/PI+).

**Western blot analysis**

The cells treated with different conditions were collected and lysed with RIPA cell

lysate, and the protein was collected after centrifugation at 12000 rpm in a centrifuge, and the protein was quantified by the BCA method. The same amount of protein in each group was separated by electrophoresis, electrotransfer to PVDF membrane, blocked with 5% skim milk for one hour, the primary antibody was incubated overnight, and the secondary antibody was incubated at room temperature for one hour. The protein expression level was detected by Enhanced chemiluminescence (ECL) detection kit.

**Quantitative RT-PCR**

Cells treated with different conditions were harvested, RNA was extracted by RNA isolation kit (Tiangen, Beijing, China) and cDNA was synthesized with Goldenstar™ RT6 cDNA Synthesis Kit (Tsingke, Beijing, China). Real-time PCR reactions were performed on Applied Biosystem 7300 real time PCR system using SYBR Green PCR Master Mix (Yeasen, Shanghai, China). Primer pairs are as follows. The forward and reverse primers of c-Fos were 5′-TTCAACGCAGACTACGAGGC-3′ and 5′-TGAAGTTGGCACTGGAGACG-3′. The forward and reverse primers of GAPDH were 5′-GGAGCGAGATCCCTCCAAAAT-3′and 5′-GGCTGTTGTCATACTTCTCATGG-3′.

**Chromatin immunoprecipitation (ChIP) assay**

The ChIP assay was performed using a CUT&RUN Assay Kit (Cell Signaling Technology, Inc.), according to the manufacturer’s protocol. U2932 cells were treated with different concentrations of LAQ824(0μM,0.01μM,0.1μM) for 24h.Then cells were harvested, washed, bound to concanavalin A-coated magnetic beads and permeabilized. After that, the bead-cell complex was incubated overnight at 4°C with rabbit IgG antibody (66362, Cell Signaling Technologies), rabbit anti-Tri-Methyl-Histone H3 (Lys4) (C42D8) antibody (9751, Cell Signaling Technologies), rabbit anti-H3K9ac antibody (A7255, ABclonal,3ug), rabbit anti-pH3Ser10(701258, Thermo Fisher Scientific, 3 µg). The DNA was extracted by DNA Purification Buffers and Spin Columns (#14209, Cell Signaling Technology) and detected by qRT-PCR. Enrichment was calculated as follows: % of input = 2 ^ (Ct input – Ct ChIP) × 100%. The immunoprecipitated DNA was analyzed by polymerase chain reaction (PCR) using the following primers:

-1000 c-Fos F:5′-CTTGTCATCCCGAACTGACC-3′;

-1000 c-Fos R:5′-AATCCCTGAGACACCAGCAG-3′;

-300 c-Fos F:5′-GAGCAGTTCCCGTCAATCC-3′;

-300 c-Fos R:5′-GCATTTCGCAGTTCCTGTCT-3′;

+150 c-Fos F:5′-TACCCAGCTCTGCTCCACA-3′;

+150 c-Fos R:5′-GTCTGCGGGTGAGTGGTAGT-3′;

+1000 c-Fos F:5′-TGGCAGGATCGTTTCTCTTC-3′;

+1000 c-Fos R:5′-GTGGGAATGAAGTTGGCACT-3′;

+2000 c-Fos F:5′-AAGGGGAAAGTTGGAGATTGA-3′;

+2000 c-Fos R:5′-GTGAGCTGCCAGGATGAACT-3′;

+3000 c-Fos F:5′-GCCTGGGTCTGTGTCTCTTT-3′;

+3000 c-Fos R:5′-GAACACACTATTGCCAGGAACA-3′;

GAPDH F:5′-GGAGCGAGATCCCTCCAAAAT-3′;

GAPDH R:5′-GGCTGTTGTCATACTTCTCATGG-3′.

**Human apoptosis signaling array**

Human Apoptosis Signaling Pathway Array C1 (RayBiotech, USA) was used to analyze expression and activation of apoptotic proteins. DLBCL cells were treated for 24 h with vehicle or LAQ824.Then the Cell Lysate were collected and quantified using BCA protein assay. The protein array membranes were loaded equal amounts of protein, blocked, incubated and washed according to the manufacturer’s instructions. Finally, membranes were visualized by ImageQuant LAS4000 Scanner (GE Healthcare Corporate, USA) and analyzed by AAH-APOSIG-1 software.

**siRNA transfections**

The electrotransfection was conducted at 180V by BIO-RAD Gene Pulser Xcell Electroporation System for suspension cells(U2932). siRNAs were ordered as HPLC-purified duplexes from KeyGEN Biotech (KeyGEN, Nanjing, China). The sequences of the siRNAs used in this study are shown as follows.

c-Fos si-1 sense:5′-GCAAGGUGGAACAGUUAUCTT-3′;

c-Fos si-1 antisense:5′-GAUAACUGUUCCACCUUGCTT-3′;

c-Fos si-2 sense:5′-AGGAGAAUCCGAAGGGAAATT-3′;

c-Fos si-2 antisense:5′- UUUCCCUUCGGAUUCUCCUTT-3′.

**messenger RNA (mRNA)-sequencing**

mRNA sequencing performed by Geneseed Biotech (Guangzhou, China)was conducted for gene expression profiling of DLBCL cell lines treated with LAQ824. RNA‐Seq reads were mapped to the updated UCSC transcript set using Bowtie2 version 2.1.0^1^. The gene expression level was estimated using RSEM v1.2.15^2^ and normalized by TMM (trimmed mean of M-values). The edgeR program was used to identify differentially expressed genes^3^.The altered expression level of genes with p < 0.05 and more than 1.5 fold changes were considered differentially expressed. Ingenuity (IPA) was applied to perform the pathway and network analysis and compute a score for each network according to the fit of the set of supplied focus genes^4^.These scores indicate the possibility of focus genes to belong to a network versus those acquired accidentally. A score > 2 indicates that a focal gene network was not generated by chance alone. The canonical pathways generated by IPA are the most significant for the uploaded data set. The significance of the canonical pathway was calculated by Fischer’s exact test with FDR option.

**Whole exome sequencing**

The exome sequences were efficiently enriched from 0.4 μg genomic DNA using Agilent liquid capture system (Agilent SureSelect Human All Exon V6) according to the manufacturer’s protocol. Products were purified using AMPure XP system (Beckman Coulter, Beverly, USA), DNA concentration was measured by Qubit®3.0 Flurometer (Invitrogen, USA), libraries were analyzed for size distribution by NGS3K/Caliper and quantified by realtime PCR (3 nM). At last, DNA library were sequenced on Illumina for pairedend 150bp reads. An adaptor-ligated library was prepared with the Paired-End Sample Prep kit V1 (Illumina). Exome capture was performed with the SureSelect Human All Exon V6 (Agilent Technologies). Massively parallel sequencing was performed on a HiSeq 2500 (Illumina) in a pair-end 150bp mood.

**Whole exome sequencing analysis**

Sequence artifacts, including reads containing adapter contamination, low-quality nucleotides and unrecognizable nucleotide (N), undoubtedly set the barrier for the subsequent reliable bioinformatics analysis. Hence quality control is an essential step and applied to guarantee the meaningful downstream analysis. Valid sequencing data was mapped to the reference human genome (GRCh38) by Burrows Wheeler Aligner (BWA) software to get the original mapping results stored in BAM format^5^. Then, SAMtools and samblaster were used to sort BAM files and do duplicate marking, to generate final BAM file for computation of the sequence coverage and depth^6^. GATK (https://gatk.broadinstitute.org/hc/en-us)were used to do variant calling and identify SNP, InDels. The somatic SNV and InDel was detected by muTect, and Control-FREEC was used to detect somatic CNV^7, 8^.ANNOVAR is performed to do annotation for VCF (Variant Call Format) file obtained in the previous step^9^. The variant position, variant type, conservative prediction and other information are obtained at this step through a variety of databases.

**Single cell RNA sequencing**

Single-cell suspensions with 1×10^5^ cells/mL in concentration in PBS (HyClone) were prepared. Single-cell suspensions were then loaded onto microfluidic devices and scRNA-seq libraries were constructed according to Singleron GEXSCOPE^®^ protocol by GEXSCOPE^®^ Single-Cell RNA Library Kit (Singleron Biotechnologies) ^10^. Individual libraries were diluted to 4nM and pooled for sequencing. Pools were sequenced on Illumina HiSeq X with 150 bp paired end reads.

**scRNA-seq quantifications and statistical analysis**

Raw reads were processed to generate gene expression profiles using an internal pipeline. Briefly, after filtering read one without poly T tails, cell barcode and UMI was extracted. Adapters and poly A tails were trimmed (fastp V1) before aligning read two to GRCh38 with ensemble version 92 gene annotation (fastp 2.5.3a and featureCounts 1.6.2) ^11^. Reads with the same cell barcode, UMI and gene were grouped together to calculate the number of UMIs per gene per cell. The UMI count tables of each cellular barcode were used for further analysis. Cell type identification and clustering analysis using Seurat program^12, 13^.The Seurat program (http://satijalab.org/seurat/, R package,v.3.0.1) was applied for analysis of RNA-Sequencing data. UMI count tables were loaded into R using read.table function. Then we set the parameter resolution to 0.6 for FindClusters function to clustering analyses. Differentially expressed genes (DEGs) between different samples or consecutive clusters were identified with function FindMarkers. GO function enrichment analysis was performed on the gene set using the clusterProfiler software to find biological functions or pathways that are significantly associated with the genes specifically expressed ^14^.

**Supplementary Figures**

**Supplemental Figure 1 The activities of chidamide and LAQ824 in diffuse large B-cell lymphoma (DLBCL) cells.**

**(A, B)** The cytotoxicity of the LAQ824 and chidamide was investigated in vitro in different DLBCL cell lines.

**Supplemental Figure 2 Single-cell RNA-Seq analysis of diffuse large B-cell lymphoma (DLBCL) cells treated with LAQ824**

1. Heatmap showing the expression of the top 10 marker genes for each sample treated with various drug concentrations. The color bar is shown in Figure 2B. **(B)** Differential gene expression levels of each sample are represented with a dot plot. **(C)** Heatmap of cluster signature genes of top differentially expressed genes (rows) across the cells (columns) in each cluster (color bar, top). **(D)** tSNE visualization of the selected gene expression. **(E)** tSNE plot showing the expression of CD79A, MS4A1, MZB1, and HLA-DRA expression. **(F)** Frequency plots of genome copy number variations (CNVs) for different concentrations of LAQ824. Red indicates CNV gain and blue CNV loss.

**Supplemental Figure 3 The expression of c-Fos was elevated in diffuse large B-cell lymphoma (DLBCL) cells treated with LAQ824.**

1. Volcano plot showing differentially expressed genes. (B) Expression levels of c-Fos in different samples. **(C)** The expression levels of c-Fos in each cluster are shown by a violin plot. **(D**) We performed Bulk RNA-sequencing of different concentrations (0/0.01/0.05/0.1 μM) of LAQ824 to delineate gene expression heterogeneity. Three biological replicates were used for each condition.

**Supplemental Figure 4 Single-cell RNA-Seq analysis of cluster with overexpression of c-Fos.**

**(A)** The t-SNE plot of diffuse large B-cell lymphoma (DLBCL) cells treated with LAQ824 0.1 μM. Cells are colored according to the different cell subpopulations**. (B)** Proportion and distribution of cells in each subset. The color bars are shown in Supplemental Figure 4A. **(C)** Dot plot showing the differential gene expression of each subpopulation in DLBCL cells treated with LAQ824 0.1 μM. **(D)** Expression of top differentially expressed genes (rows) across the cells (columns) treated with LAQ824 at 0.1 μM of ten subclusters. (color bar, top) **(E)** t-SNE of DLBCL cells treated with LAQ824 0.1 μM colored by expression of key cell-type marker genes. **(F)** Distribution of c-Fos expression levels across the 10 subclusters. The color bar is shown in Supplemental Figure 4A. **(G)** Pseudotime and single-cell trajectory analysis of DLBCL cells treated with LAQ824 at 0.1 μM of Monocle. **(H)** Monocle trajectory visualization for each cluster. **(I)** The expression patterns of marker genes are plotted along the pseudo time axis.

**Supplemental Figure 5 The action of other drugs in diffuse large B-cell lymphoma (DLBCL).**

1. IC50 values of DLBCL cell lines with ABC or GCB DLBCL were analyzed. ns, no significance. **(B)** Synergistic cell-killing effect of LAQ824 and c-Fos inhibitor (T-5224) combination treatment. **(C)** qRT-PCR detected the mRNA expression level of c-Fos in the DLBCL cell line (U2932) treated with different inhibitors. Data are represented as mean ± SD. * p < 0.05, ** p < 0.01, *** p < 0.001 versus the control group.

**Supplementary Tables**

**Table S1 Combination Index (CI) of c-Fos inhibitors (T-5224 and CDF) with LAQ824 in each DLBCL cell line.**

| Cell lines | LAQ824 (μM) | Incubation time (h) | CDF (0.01μM) | T-5224 (10μM) |
| --- | --- | --- | --- | --- |
| U2932 | 0.01 | 24 | 0.94292 | 1.018444 |
|  |  | 48 | 0.9018 | 1.030939 |
|  |  | 72 | 0.96877 | 1.06311 |
|  | 0.1 | 24 | 0.36538 | 0.74598 |
|  |  | 48 | 0.05046 | 0.30787 |
|  |  | 72 | 0.07726 | 0.0709 |
| HBL-1 | 0.01 | 24 | 0.99428 | 1.03337 |
|  |  | 48 | 0.93296 | 0.9135571 |
|  |  | 72 | 0.8818 | 0.97889 |
|  | 0.1 | 24 | 0.54178 | 0.46983 |
|  |  | 48 | 0.10965 | 0.70461 |
|  |  | 72 | 0.10974 | 0.62585 |
| CTB-1 | 0.01 | 24 | 1.00313 | 0.973446 |
|  |  | 48 | 0.86561 | 0.882146 |
|  |  | 72 | 0.94122 | 1.00784 |
|  | 0.1 | 24 | 0.01374 | 0.2161 |
|  |  | 48 | 0.00655 | 0.33189 |
|  |  | 72 | 0.06845 | 0.05251 |
| Farage | 0.01 | 24 | 1.05522 | 0.9121918 |
|  |  | 48 | 0.91148 | 0.9148452 |
|  |  | 72 | 0.98277 | 0.8914966 |
|  | 0.1 | 24 | 0.69487 | 0.7344 |
|  |  | 48 | 0.38319 | 0.69052 |
|  |  | 72 | 0.33422 | 0.67443 |

**Table S2 The quantification of the different protein spots in protein array analysis.**

| **proteinID** | **AveExp.LAQ0** | **AveExp.LAQ0.1** | **logFC** | **foldchange** | **regulation** | **entrezID** | **uniprotID** | **LAQ0** | **LAQ0.1** | **Threshold** |
| --- | --- | --- | --- | --- | --- | --- | --- | --- | --- | --- |
| PARP | 11.26781082 | 11.00433798 | 0.263472842 | 1.200364736 | up | 142 | P09874 | 2464.75 | 2053.167309 | TRUE |
| IkBa (S32) | 10.08115052 | 9.778964709 | 0.302185813 | 1.233011121 | up | 4792 | P25963 | 1082.25 | 877.540332 | TRUE |
| SMAD2 | 10.33231633 | 10.01106366 | 0.321252672 | 1.249414928 | up | 4087 | Q15796 | 1288.25 | 1030.882981 | TRUE |
| Erk1/2(t202) | 11.03840463 | 10.67737639 | 0.361028235 | 1.284340945 | up | 5594 | P28482 | 2102.25 | 1636.610331 | TRUE |
| hsp27(S82) | 10.09967655 | 9.727864894 | 0.371811661 | 1.293976719 | up | ?3315 | P04792 | 1096.25 | 846.9673427 | TRUE |
| eIF2a (S52) | 11.90096112 | 11.52666456 | 0.374296568 | 1.296207393 | up | 83939 | Q9BY44 | 3823.25 | 2949.338056 | TRUE |
| ATM | 11.39914438 | 11.02068467 | 0.378459712 | 1.299953224 | up | 472 | Q13315 | 2699.75 | 2076.574754 | TRUE |
| AKT(S473) | 11.77961677 | 11.36961985 | 0.409996921 | 1.328682979 | up | 207 | P31749 | 3514.75 | 2645.041273 | TRUE |
| TAK1 | 11.44747072 | 11.00198754 | 0.445483177 | 1.361770115 | up | 6885 | O43318 | 2791.75 | 2049.823388 | TRUE |
| P53(S15) | 11.36673201 | 10.87379921 | 0.4929328 | 1.407302822 | up | 6198 | P23443 | 2639.75 | 1875.461809 | TRUE |
| CHK1 (S280) | 12.13947141 | 11.56014412 | 0.579327296 | 1.494152388 | up | 1111 | O14757 | 4510.75 | 3018.604985 | TRUE |
| P27(T198) | 11.19028768 | 10.57266387 | 0.617623808 | 1.534345953 | up | 5715 | O00233 | 2335.75 | 1521.961621 | TRUE |
| P38(T180/Y182) | 12.49878689 | 11.41777312 | 1.081013775 | 2.115522126 | up | 1432 | Q16539 | 5786.75 | 2734.849429 | TRUE |
| CHK2(T68) | 13.3257622 | 11.61192591 | 1.71383629 | 3.280319389 | up | 11200 | O96017 | 10266.25 | 3128.954368 | TRUE |

**References**

1. Langmead B, Salzberg SL. Fast gapped-read alignment with Bowtie 2. *Nat Methods* 2012 Mar 4; **9**(4)**:** 357-359.

2. Li B, Dewey CN. RSEM: accurate transcript quantification from RNA-Seq data with or without a reference genome. *BMC Bioinformatics* 2011 Aug 4; **12:** 323.

3. Robinson MD, McCarthy DJ, Smyth GK. edgeR: a Bioconductor package for differential expression analysis of digital gene expression data. *Bioinformatics* 2010 Jan 1; **26**(1)**:** 139-140.

4. Kramer A, Green J, Pollard J, Jr., Tugendreich S. Causal analysis approaches in Ingenuity Pathway Analysis. *Bioinformatics* 2014 Feb 15; **30**(4)**:** 523-530.

5. Li H, Durbin R. Fast and accurate short read alignment with Burrows-Wheeler transform. *Bioinformatics* 2009 Jul 15; **25**(14)**:** 1754-1760.

6. Tarasov A, Vilella AJ, Cuppen E, Nijman IJ, Prins P. Sambamba: fast processing of NGS alignment formats. *Bioinformatics* 2015 Jun 15; **31**(12)**:** 2032-2034.

7. Boeva V, Popova T, Bleakley K, Chiche P, Cappo J, Schleiermacher G*, et al.* Control-FREEC: a tool for assessing copy number and allelic content using next-generation sequencing data. *Bioinformatics* 2012 Feb 1; **28**(3)**:** 423-425.

8. Cibulskis K, Lawrence MS, Carter SL, Sivachenko A, Jaffe D, Sougnez C*, et al.* Sensitive detection of somatic point mutations in impure and heterogeneous cancer samples. *Nat Biotechnol* 2013 Mar; **31**(3)**:** 213-219.

9. Wang K, Li M, Hakonarson H. ANNOVAR: functional annotation of genetic variants from high-throughput sequencing data. *Nucleic Acids Res* 2010 Sep; **38**(16)**:** e164.

10. B D, JY C, K Z, W D, D T, M B*, et al.* scFTD-seq: freeze-thaw lysis based, portable approach toward highly distributed single-cell 3' mRNA profiling. *Nucleic acids research* 2019; **47**(3)**:** e16.

11. Y L, GK S, W S. featureCounts: an efficient general purpose program for assigning sequence reads to genomic features. *Bioinformatics (Oxford, England)* 2014; **30**(7)**:** 923-930.

12. R S, JA F, D G, AF S, A R. Spatial reconstruction of single-cell gene expression data. *Nature biotechnology* 2015; **33**(5)**:** 495-502.

13. A B, P H, P S, E P, R S. Integrating single-cell transcriptomic data across different conditions, technologies, and species. *Nature biotechnology* 2018; **36**(5)**:** 411-420.

14. G Y, LG W, Y H, QY H. clusterProfiler: an R package for comparing biological themes among gene clusters. *Omics : a journal of integrative biology* 2012; **16**(5)**:** 284-287.
